# Effect of the Narcissism Subscale “Threatened Self” on the Occurrence of Burnout Among Male Physicians

**DOI:** 10.3390/jcm14103330

**Published:** 2025-05-10

**Authors:** Antonia Tiziana Kreis, Roland von Känel, Sarah Andrea Holzgang, Aju Pazhenkottil, Jeffrey Walter Keller, Mary Princip

**Affiliations:** 1 Department of Consultation-Liaison Psychiatry and Psychosomatic Medicine, University Hospital Zurich, University of Zurich, 8091 Zurich, Switzerland; 2 Cardiac Imaging, Department of Nuclear Medicine, University Hospital Zurich, 8091 Zurich, Switzerland; 3 Department of Cardiology, University Hospital Zurich, 8091 Zurich, Switzerland

**Keywords:** narcissism, threatened self, burnout, physicians, job stress

## Abstract

**Background/Objectives:** Burnout is a highly prevalent issue among physicians. Recent research has indicated that personality traits, such as narcissism, may influence the development of burnout. This study investigates the relationship between the threatened self (TS) narcissism subscale and burnout in male physicians. **Methods:** We analyzed data from 60 male physicians in Switzerland, divided into burnout (*n* = 30) and control (*n* = 30) groups. Male physicians in Switzerland were recruited via hospitals, clinics, medical associations, professional journals, and direct email outreach. We assessed participants using the Maslach burnout inventory (MBI-HSS) and the Narcissism Inventory (NI-20). A generalized linear model (GLM) was used for the statistical analysis. **Results:** The results showed that lower TS scores were significantly associated with a reduced likelihood of burnout, suggesting that self-esteem instability and emotional vulnerability, characteristic of TS, may act as risk factors for burnout. Furthermore, we found that Effort–Reward Imbalance (ERI) was significantly associated with burnout. **Conclusions:** These findings highlight the importance of considering personality traits such as TS in burnout research and could be explored in further studies. In clinical practice, increasing therapists’ awareness of TS may support more targeted interventions and help prevent the onset of burnout.

## 1. Introduction

The increasing presence of the concept of burnout in societal discourses has led to a growing scientific interest in recent decades. Burnout is defined as a negative affective risk state for the development of mental and physical consequences and is characterized by three core symptoms, which are assessed with the Maslach burnout inventory (MBI) [1]: emotional exhaustion (EE), depersonalization (DP), and reduced personal accomplishment (PA) in terms of professional competencies [2]. Physicians are especially strongly affected by burnout, with an increasing trend in recent years [3,4]. Burnout negatively impacts both one’s personal and professional well-being [5,6,7,8,9,10,11]. Among physicians, burnout is linked to compromised professionalism, a heightened risk of medical errors, diminished care quality [11], decreased patient satisfaction, and longer post-discharge recovery time in patients [12,13], as well as reduced career and work satisfaction [14,15]. Regarding the prevalence of burnout among Swiss physicians, a 2023 survey by the Association of Swiss Junior and Senior Hospital Doctors (VSAO) highlights a concerning decline in physician well-being. Indicators of poor health have worsened over the previous three years: fatigue, emotional and physical exhaustion, and feelings of being overwhelmed have shown a marked increase among resident and senior physicians [16]. Further research shows an increasing prevalence of burnout among physicians in Switzerland [3], with younger physicians appearing to be particularly vulnerable [17].

While early research on burnout emphasized work-related factors, recent studies have increasingly recognized the role of personality characteristics in how individuals respond to different work situations, potentially increasing the risk of burnout [18,19]. A recent study identified a significant link between social comparison rumination and core components of burnout in students, particularly emotional exhaustion and cynicism. These findings underscore the potential role of maladaptive cognitive processes in the development of burnout. People with an overly idealized self-image may react strongly when others seem more successful or popular. This can lead to ongoing preoccupation and rumination about these social comparisons [20]. Among other personality traits, the impact of narcissism on burnout has been discussed. Before examining the role of narcissism in burnout, some general aspects of narcissism should be outlined, including the distinction between healthy and pathological forms, and with specific attention to the threatened self (TS), an important subdimension of narcissism.

Narcissism reflects an individual’s ability to maintain a positive self-image through various cognitive, emotional, and behavioral regulatory processes [21]. This empowers the individual to seek validation, confirmation, and experiences of self-enhancement from the social environment [22]. Narcissism, as a regular aspect of personality, includes qualities such as high self-centeredness, a sense of superiority, a longing for admiration of others, and a tendency toward socially incompatible behaviors. A grandiose yet vulnerable self-concept explains the constant pursuit of external validation. The strong self-centeredness, coupled with a simultaneous lack of interest in others who are perceived as inferior, ultimately hinders the positive feedback that narcissistic personalities pursue [23]. The terms narcissism and narcissistic personality disorder have been used interchangeably for a long time. In recent times, the term narcissism has evolved to denote a personality variable in healthy individuals and should be distinguished from narcissistic personality disorder [24]. Differentiating precisely between healthy and pathological narcissism remains a challenge [25,26]. There are various approaches to describing and categorizing narcissism. Personality psychology distinguishes between grandiose and vulnerable manifestations of narcissistic traits. The transition from normative to pathological narcissism is marked by an increasing predominance of vulnerable characteristics [27].

The concept of the ‘Threatened Self’ (TS) was first introduced as a subdimension of narcissism in the original Narcissism Inventory (NI) developed by Deneke and Hilgenstock (1989) [28]. In its revised short form, the Narcissism Inventory-20 (NI-20), which is applied in the present study, narcissism is conceptualized along four dimensions, including the TS subscale [29]. These subscales are further described in detail in the following Methods section. TS reflects self-regulatory processes ranging from structural cohesion to narcissistic decompensation, indicating highly unstable self-esteem [28]. Despite its conceptual importance, research on the TS dimension is limited. In particular, the potential link between TS and burnout remains largely unexplored, highlighting a notable gap in the literature. TS represents an interesting aspect, particularly regarding underlying theories of the self. According to Claude Steele’s (1988) [30] Self-Affirmation Theory, individuals are motivated to maintain a self-image that is coherent, morally adequate, and competent. When this self-image is threatened by failure, criticism, or social devaluation, for instance, it can trigger psychological defense mechanisms to protect the perceived integrity of the self [30]. This mechanism becomes particularly significant in the context of unstable self-esteem. Unstable self-esteem is considered a vulnerable predictor of maladaptive responses to self-threatening situations [31]. Individuals with unstable self-esteem are more prone to defensive reactions, such as externalizing blame or avoiding the source of threat. Coping with self-threat has also been investigated in the field of neuroscience; an fMRI study revealed that individuals with high self-esteem engage more strongly in self-enhancing strategies following a threat by more effectively inhibiting negative self-related information compared to those with low self-esteem [32]. Previous research has already identified a significant and positive association between narcissism and burnout and examined various aspects of this relationship [19,33,34,35]. There has been research on narcissism and burnout, exploring the subscales of adaptive and maladaptive narcissism [19] and research investigating grandiose narcissism, specifically admiration and rivalry in relation to burnout [36]. Schwarzkopf et al. (2016) examined the relationship between narcissistic personality traits and job burnout among 723 hospitalized patients with job-stress-related disorders, showing a significant correlation [34]. This study also examined the relationship between burnout and the four narcissism subscales of classic narcissistic self (CnS), idealistic self (IS), hypochondriac self (HS), and threatened self (TS), which are also employed in the present study. Rana et al. (2022) [35] examined the relationship between narcissism and burnout in a sample of surgeons in Germany, focusing on two dimensions of narcissism: “Admiration” and “Rivalry”. High rivalry scores were linked to higher levels of burnout [35]. Other findings, comparing grandiose narcissism among surgeons, indicate that female participants exhibit significantly lower levels of narcissistic rivalry compared to their male colleagues [37]. This is an important finding pertaining to the present study, as our investigation was limited to male physicians. This decision has further reasons related to the study design, which are explained in more detail in the methodology section. Another recent study by Klerks et al. (2024) [38] examining the relationship between the dark triad (Machiavellianism, vulnerable and grandiose narcissism, and psychopathy) and burnout, mediated by perfectionistic self-presentation, found significant positive correlations between grandiose narcissism, academic burnout, and the Perfectionistic Self-Presentation Scale (PSPS). This study revealed that the relationship between grandiose narcissism and academic burnout was significantly mediated by the non-display of imperfections [38]. Burnout and narcissism among physicians have been explored to a limited extent, with the previously discussed study from 2022 specifically investigating this relationship in surgeons [35]. However, we further specify this research question by focusing directly on the TS narcissism subscale. While there has been existing research on narcissism and burnout among other study populations, as well as specifically on the TS subscale and burnout, the combination of these two components—the study population of physicians and TS—brings novelty to the field. Narcissism has been explored extensively; the TS subscale, which demonstrated the most significant associations with burnout compared to other dimensions in previous research [34], remains an area of untapped potential. Notably, there has been no prior investigation into the correlation between the TS subscale and physicians to our knowledge. In the present study, we examined the relationship between the TS narcissism subscale and burnout among physicians, exploring whether a high score of TS acts as a predictor for burnout.

Our hypothesis posits that TS serves as a positive predictor for the occurrence of burnout. The aim of this study is to gain a deeper understanding of this relationship, which may help identify a potential at-risk group and support its early recognition in the future. In addition, our findings may contribute to raising awareness of the influence of narcissistic personality traits on the development of burnout, highlighting possible clinical and therapeutic implications, and ultimately promoting a healthier workplace culture. By investigating TS as a potential predictor, this study addresses a notable gap in current research, as this subdimension of narcissism has rarely been examined in physician populations despite its theoretical relevance. As outlined above, TS plays a particularly important role in the psychological mechanisms underlying vulnerability to burnout, making it a compelling focus for further exploration.

## 2. Materials and Methods

### 2.1. Design

The following study is a secondary analysis of a previously conducted cross-sectional study that focused on the cardiovascular health of male physicians experiencing burnout [39]. We collected data between September 2019 and December 2021. Participation in this study was entirely voluntary, and we obtained informed consent from all participants. We recruited male physicians in Switzerland by contacting them through various channels, including hospitals, clinics, medical associations, professional publications, and direct email communication. Inclusion criteria were male physicians between 28 and 65 years of age, non-smokers for at least five years, and clinical burnout for the burnout group or absence of burnout for the control group based on the specified criteria. Exclusion criteria included any prior episode of clinical depression or burnout, current depression, cognitive impairment, a history of known heart disease, familial hypercholesterolemia, renal insufficiency, hypertension, diabetes, obesity, allergies to iodinated contrast media, contraindications to adenosine, beta-blockers, or isosorbide dinitrate, as well as a preference not to receive information regarding clinically relevant cardiac assessments. We included only male physicians based on the strict inclusion criteria of the parent study “Coronary microvascular function in male physicians with burnout and job stress”. The parent study focused on working-age men (28–65 years) because the association between work stress and coronary heart disease has consistently been demonstrated in this group. In contrast, findings in women remain inconclusive, likely due to insufficient evidence and the fact that cardiovascular disease in women often manifests later in life, frequently beyond the typical working age [40]. Moreover, male physicians show a markedly higher rate of major adverse cardiovascular events than their female colleagues [41]. Finally, this decision aimed to minimize potential confounding factors, particularly hormonal influences on various blood biomarkers that were among the measures assessed. We included a total of 60 male participants in the study.

### 2.2. Ethics

This research project obtained approval from the local ethics committee of the State of Zurich, Switzerland (BASEC-Nr. 2018-01974). All participants provided written informed consent.

### 2.3. Participants

We included a total of 60 male participants in this study. These 60 participants were divided into two groups, consisting of 30 individuals each in the burnout and healthy control groups. We used the Maslach burnout inventory Human Services Survey (MBI-HSS; Mind Garden, Inc., Menlo Park, CA, USA) [42] and the Patient Health Questionnaire (PHQ-9; Pfizer Inc., New York, NY, USA) [43] to determine group assignments, conducting these surveys during the phone screening. To ensure a clear distinction between the two groups, we established cutoff values based on the review by Rotenstein et al. [44]. For the burnout group, the following cutoffs for the MBI-HSS were used: EE ≥ 27 and/or DP ≥ 10 (with a minimum EE of ≥20). For the healthy control group, the criteria were EE < 16 and DP < 7 [44]. We did not use the personal accomplishment (PA) subscale for group assignment, as previous research has shown that PA is relatively independent of the other two subscales [45,46,47]. In the case of the burnout group, a PHQ-9 score of ≤14, indicating at most moderate depressive symptoms, was required. For the healthy control group, a PHQ-9 score of ≤10, reflecting at most mild depressive symptoms, was necessary [48].

The total sample size was determined for the purpose of the parent study “Coronary microvascular function in male physicians with burnout and job stress” (Von Känel et al., 2023) [39], which involved extensive PET-based myocardial imaging. The decision was informed by previous institutional research conducted in patients with a sleep disorder [49], which indicated that a sample size of 23 participants per group was sufficient to detect a large effect (Cohen’s d = 0.85) in adenosine-induced hyperemic myocardial blood flow, with 80% statistical power. To ensure adequate power and account for potential data loss, we aimed to enroll a total of 60 participants. Due to the complexity and resource-intensity of these procedures, a larger sample was not feasible.

### 2.4. Instruments

Participants completed the questionnaires NI20, ERI, and PSS-4 using printed forms on the day of the examination. For the MBI, all responses were taken from the examination day, except for one participant, for whom all MBI responses were missing and were therefore obtained from the initial phone screening.

Maslach burnout inventory (MBI): The MBI is a self-evaluation questionnaire used to assess the severity of burnout [1]. Our study used the 22-item German adaptation of the MBI-Human Services Survey [42]. Each of the 22 items is rated on a 7-point scale ranging from 0 (“never”) to 6 (“daily”). These 22 items constitute the three dimensions of burnout: “Emotional exhaustion” (EE, nine items), “Depersonalization” (DP, five items), and “Personal achievement” (PA, eight items). The EE dimension examines feelings of being emotionally overwhelmed and exhausted because of work, while the DP dimension assesses a detached and impersonal response towards care recipients or patients. The PA dimension examines feelings of competence and successful accomplishments in one’s work. Each of these dimensions can be evaluated independently. In this study, we identified a strong internal consistency for the EE dimension (Cronbach’s α = 0.94), as well as a good internal consistency for the DP dimensions (Cronbach’s α = 0.89) and PA (Cronbach’s α = 0.81).

Narcissism Inventory (NI-20): We used the 20-item short version of the self-reported Narcissism Inventory (NI-20) to measure narcissistic aspects [29]. The NI-20 items reflect a variety of narcissistic characteristics. As discussed by Deneke and Hilgenstock in 1989 [28], narcissistic self-regulation can be divided into four dimensions: threatened self (TS, seven items), classic narcissistic self (CnS, five items), idealistic self (IS, four items), and hypochondriac self (HS, four items). The TS dimension reflects self-organization ranging from cohesion to narcissistic decompensation, indicating unstable self-esteem. TS comprises 8 subscales, such as helpless self, loss of control over affects and impulses, derealization/depersonalization, basic potential of hope, worthless self, negative bodily self, social isolation, and withdrawal into feelings of harmony [29]. CnS corresponds to the traditional facet of narcissistic personality, emphasizing egocentrism or an inflated sense of one’s abilities, reflecting aspects of narcissistic traits. The IS dimension addresses latent or overt anxiety about potential disappointment or emotional wounds in relationships, manifested through attempts to stabilize oneself by identifying with idealized models. HS, the fourth dimension, evaluates attention to one’s own body and how it is perceived and utilized as an object, representing a hypochondriac anxiety-binding mode of self-regulation [29,50]. Participants assessed each item using a 5-point Likert scale, with options ranging from 1 (not applicable at all) to 5 (fully applicable). In our sample, the internal consistency of the dimension TS was Cronbach’s α = 0.696.

Perceived Stress Scale (PSS-4): We employed the 4-item German version of the PSS-4 to assess the extent to which individuals perceived situations as stressful during the past month [51]. This short version has demonstrated good internal consistency in a validation study [52]. Using a 5-point Likert scale, ranging from 0, representing “never”, to 4, indicating “very often”, questions were answered. A higher score on the PSS-4 signifies a higher level of perceived stress. In our sample, the total score showed an excellent internal consistency, with Cronbach’s α = 0.86.

Effort–Reward Imbalance (ERI): We evaluated job-related stress using the German short form of the Effort–Reward Imbalance questionnaire (ERI; University of Düsseldorf, Düsseldorf, Germany). The questionnaire includes three items assessing the effort put into work, seven about the rewards obtained at work, and six items measuring overcommitment [53]. Each item is rated on a 4-point Likert scale, ranging from 1, indicating ‘strongly disagree’, to 4, indicating ‘strongly agree’. We calculated the effort–reward ratio while considering a correction factor to address the differing number of effort and reward items. A higher ratio signifies higher job stress. In our sample, the internal consistency for the effort scale showed a Cronbach’s α of 0.76 and 0.77 for the reward scale. This German-language version also demonstrated satisfactory psychometric properties in a validation study, supporting its continued use in research and practice [54]. In two studies conducted among physicians in Switzerland, the ERI questionnaire was applied, and the validity for use in this context was demonstrated [55,56].

### 2.5. Statistical Analysis

The present analysis represents a secondary analysis derived from the parent study “Coronary microvascular function in male physicians with burnout and job stress”. We performed statistical analyses using R statistical software version 4.4.1 [57]. A *p*-value below 0.05 was considered statistically significant.

Although the sample size has been predetermined, a crude post hoc sample size calculation based on the number of predictors was performed for verification purposes, using the formula *n =* 20 *+* (10 *×* predictors), which resulted in a required sample size of 60. The statistical power of our final model was assessed using the pwr package (version 1.3-0).

Group comparisons to compare demographic data were either unpaired *t*-tests or Chi-squared tests where appropriate.

A linear regression analysis was conducted. Due to a single highly influential outlier in the calculations, the analysis was performed twice: once with the outlier and once without. Model quality metrics, i.e., R squared values, were calculated using 10-fold cross-validation.

Furthermore, we used a logistic regression with the outcome burnout and the independent variable TS of the 20-item Narcissism Inventory. We tested the impact of TS on the occurrence of burnout, calculating odds ratios to quantify the strength of the association. In our model, we controlled for PSS-4, ERI, and age. As a model quality metric, we calculated the average area under the receiver operating characteristic curve (ROC AUC), sensitivity, and specificity using 10-fold cross-validation. No explicit threshold optimization was applied; thus, the predefined cut-off was left at 0.5. Finally, we calculated the classification accuracy of the model on the original data set.

## 3. Results

The demographic characteristics of the burnout group and the control group exhibited notable similarities, except for age and job satisfaction (Table 1) [58,59]. Both groups demonstrated comparable features in terms of body mass index, marital status, job status, weekly working hours, provision of night shifts, and employment status. However, physicians in the burnout group were younger (*p* = 0.012) and reported significantly lower job satisfaction (*p* < 0.001). The medical specialties were distributed as follows: approximately one-third were internists, slightly less than one-fifth were surgeons, and one-tenth were psychiatrists. There was no significant difference observed between the two groups regarding medical specialties. The two groups exhibited significant differences in the total MBI score (*p* < 0.001) the scores of the three MBI subscales (EE (*p* < 0.001), DP (*p* < 0.001), and PA (*p* < 0.001)), as expected, due to the deliberate formation of extreme groups based on burnout severity. Furthermore, the groups differed significantly in the TS narcissism subscale (*p* < 0.001), PSS-4 (*p* < 0.001), and ERI (*p* = 0.002), including all the ERI subscales (Effort subscale (*p* < 0.001), Reward subscale (*p* = 0.001), and Overcommitment subscale (*p* < 0.001) (Table 2). With an R^2^ exceeding 0.6, the model’s power was calculated to be 100%, as the explained variance was substantially higher than initially expected.

**Figure 1:**
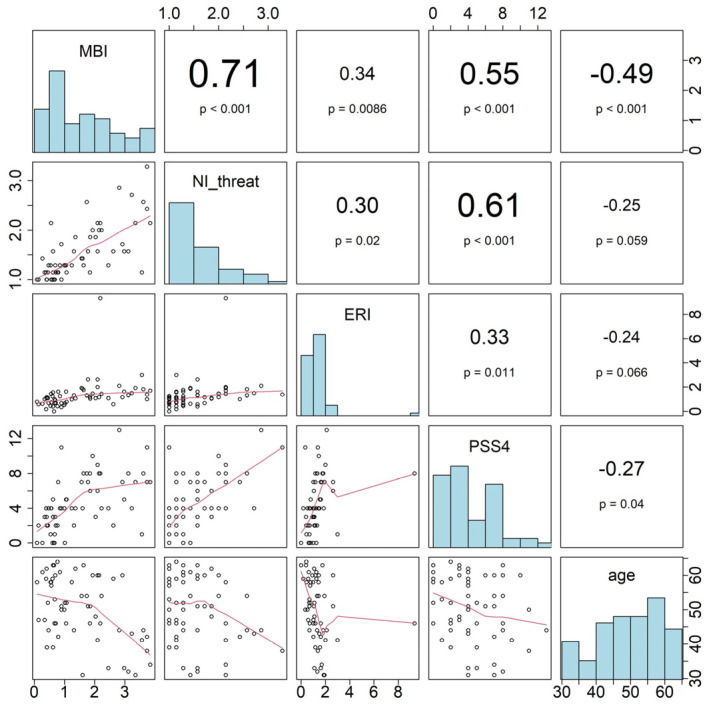
Pearson correlation of Maslach burnout inventory (MBI), Narcissism Inventory TS (NI-threat), Effort–Reward Imbalance (ERI), Perceived Stress Scale (PSS4), and age.

In the correlation matrix (Figure 1), several relationships were observed among the variables, with the strongest correlation found between MBI and TS. In the linear regression analyses, with the outlier included (Table 3) and excluded (Table 4), burnout was significantly associated with TS and age. However, in the model excluding the outlier, a significant association with Effort–Reward Imbalance (ERI) was also found. Moreover, the results indicated that a lower age was associated with higher burnout scores. Using a 10-fold cross validation, our model explains, on average, about 57% of the variance in the validation data set. Excluding the outliner, this average explained variance increases to 63%.

Using a logistic regression, Table 5 shows how a lower score on the TS narcissism subscale is significantly associated with a reduced likelihood of experiencing burnout. Controlling for PSS-4 and age, we found no significant associations, except for ERI. Like the TS, a lower ERI score is significantly associated with a reduced risk of experiencing burnout. Using 10-fold cross-validation, the model demonstrated excellent discrimination with an ROC AUC of 0.944. Sensitivity was 82%, and specificity was 80%. Applying the model to the original data set resulted in a classification accuracy of 81.4%.

## 4. Discussion

In this study, we explored the relationship between the TS narcissism subscale and burnout among a group of male physicians with burnout and a control group. Our findings indicate a significant association, suggesting that a lower score on the TS narcissism subscale is associated with a reduced likelihood of experiencing burnout. Therefore, we conclude that narcissism may function as a predictor for burnout. Our findings are consistent with previous research, which showed a significant association between burnout and narcissism [19,33,34]. Narcissism has already been assessed and investigated in various ways, using different methods and across different study populations [19,33,34,35]. The understanding and assessment of narcissism in terms of narcissistic personality disorder, pathological narcissism, and normal narcissism have been complex. There is an effort to address the challenge of reconciling inconsistent definitions of narcissism within the fields of clinical psychology, psychiatry, and social/personality psychology to capture this diverse concept within a comprehensive construct [21].

The TS narcissism dimension comprises eight subscales: helpless self, loss of control over affects and impulses, derealization/depersonalization, basic potential of hope, worthless self, negative bodily self, social isolation, and withdrawal into feelings of harmony [29]. When considering these subscales, most of the subscales entail inherently taxing and exhausting states. We can further argue that since emotional exhaustion represents a crucial dimension of burnout, such exhausting states pose a significant risk for burnout. The attributes of TS are virtually archaic, basal feelings that intuitively suggest a risk for psychological decompensation. Conversely, we know from a psychological perspective that a strong sense of self-worth, social integration, and feelings of security have a protective effect on stress experience and burnout [60,61]. The very contrasting emotional states such as “helpless self”, “worthless self”, or “social isolation” are characteristic of TS [29]. Unlike other narcissistic traits, such as the classic narcissistic self, which involves overestimating one’s abilities or seeking constant validation [29], the traits of TS pose a consistent risk for burnout due to a fundamental disruption of the self. While grandiosity fantasies may significantly impact one’s experiences and social relationships, the self is not affected as profoundly as with TS.

Focusing on the professional role of physicians, within the healthcare sector, physicians often encounter patients who exhibit neediness, hopelessness, and strong emotional burdens [62,63]. However, these patients are not threatened by a narcissistic self but rather by an illness. The symptoms of TS are reflected in affected physicians from an external source, potentially amplifying their own struggles with narcissistic structures of the threatened self, such as hopelessness or social isolation. Consequently, this situation may activate or exacerbate existing narcissistic issues and feelings of inadequacy.

Another finding of this study pertains to the relationship between ERI and burnout. Previous research shows inconsistency regarding the influence of ERI on burnout. While a significant positive impact of ERI on burnout has been demonstrated [64,65], a longitudinal cohort study found that ERI was not longitudinally associated with any of the burnout dimensions when controlling for confounders [66]. In the present study, after controlling for PSS-4 and age, it was demonstrated that a lower ERI score is significantly associated with a reduced risk of experiencing burnout. The inconclusive results across studies suggest the need for further research to explore the relationship between these components. We included participants who had to explicitly state that they had had job stress over 6 months at least, so an association would be highly expected.

Furthermore, we observed a linear association between a younger age and higher levels of burnout. This finding is consistent with the previous literature and may be attributed to lower professional experience and a resulting higher vulnerability to being overwhelmed.

In this study, the burnout group averaged 57.35 working hours per week. In contrast, non-physicians in Switzerland work an average of 41.7 h per week, with a legally regulated maximum working time of 45 h per week [67]. These additional working hours reduce opportunities for social balance and increase the risk of social isolation.

Furthermore, despite great and important efforts in recent decades to change societal stigmas, the display of weakness is traditionally viewed as a non-masculine trait [68,69]. This perception may be heightened when traits like strength, security, and the role of a harbinger of hope are projected onto physicians as a professional group. A 2020 study explored how gendered ways of thinking relate to the perception of role models in medical education. The findings revealed that male students rarely identified female doctors as role models, and male role models were generally perceived as more admirable than their female counterparts. These results highlight the ongoing influence of gendered perceptions that subtly shape the professional ideals and attitudes of medical students [70]. This can be linked to TS insofar as the mentioned masculine traits conflict with the features of TS, potentially accentuating them and thereby increasing the risk of burnout. However, this interpretation remains speculative, and further research needs to explore how men, grappling with weakened or threatened self-perceptions, possibly clash with their ideals of masculinity and the physician’s role, thereby engendering an emotionally taxing conflict, which in turn potentially heightens the risk for burnout.

Preventive measures to reduce burnout often target individual-focused interventions such as counseling, supervision, or relaxation exercises, as well as work-related interventions such as altering workflow or work organization [71]. Screening for at-risk individuals is not a practical preventive measure for narcissism. Instead, the focus can be on raising awareness among therapists working with people who are already suffering burnout, particularly in addressing aspects of the threatened self and potentially providing therapy, especially in the patient group of male physicians with burnout. TS exhibits fewer aspects of external behavior than of internal experiences, making it important to address this internal experience therapeutically. Thus, this awareness can potentially act as relapse prevention through increased diagnosis.

Our study has several noteworthy limitations. First, the sample size was relatively small (n = 60), which may reduce the statistical power and limit the generalizability of the findings. Additionally, it was divided into two predetermined extreme groups based on the severity of burnout, thereby hindering us from analyzing continuous scores of the MBI. With the ethics committee’s approval, we had to slightly relax the criteria to attain the recruitment target of 60 participants, potentially introducing methodological limitations. While we only selected male physicians for our study based on the stringent inclusion criteria of the parent study, “*Coronary microvascular function in male physicians with burnout and job stress*”, this approach aimed to minimize confounding variables, such as hormonal influences. Moreover, while we exclusively examined physicians working in Switzerland, our study population encompassed different medical specialties, enabling a broader and more generalized representation of physicians. Furthermore, since participation in our study was voluntary and contingent upon participants’ interest, we cannot disregard the possibility of both a self-selection bias and a self-reporting bias. The cross-sectional design of our study does not permit a causal conclusion regarding the association between the TS narcissism subscale and burnout. However, it can be argued that a personality variable represents a long-term condition, thus suggesting that a causal relationship can be assumed. Therefore, it is legitimate to use a predictive statistical model, such as the generalized linear model in our case. Due to the small sample size, we were not able to conduct a more detailed analysis of the eight subdimensions of the TS subscale. Such an analysis could have provided valuable insights into which specific aspects of TS have the strongest influence on burnout. Furthermore, there are several potential influencing factors, such as a history of trauma, that may impact the observed associations. These should be explored in future research. Additionally, the study was conducted during the COVID-19 pandemic. The impact of COVID-19 on the mental health of healthcare workers remains a subject of inconsistent findings in the literature. While some studies suggest that clinically relevant symptoms of anxiety and depression occurred at comparable rates before and during the pandemic [72,73], others report a marked increase in psychological distress among healthcare professionals [74]. Regarding the present study, it can be noted that this was a period when physicians faced exceptional challenges, which might have diminished their capacity to engage in this study and potentially affected their mental well-being.

## 5. Conclusions

In this study, we investigated the effect of the TS narcissism subscale on the occurrence of burnout. Our findings suggest that the increased display of the TS narcissism subscale acts as a positive predictor for experiencing burnout. However, studies with larger samples are needed to expand upon our findings. In particular, the inclusion of female physicians is warranted to examine whether the association between narcissism and burnout differs by gender. Longitudinal designs may further help to clarify causal relationships and reveal how narcissistic traits influence the development of burnout over time. Additionally, future research could explore early identification strategies and evaluate targeted therapeutic interventions for healthcare professionals exhibiting elevated levels of narcissistic traits, with the aim of preventing or mitigating burnout.

## Figures and Tables

**Table 1:** Sample characteristics.

Characteristic		Total Sample, n = 60		Burnout, n = 30				Control, n = 30					
		n (%)	Mean (SD)	n (%)	Mean (SD)	Median	IQR	n (%)	Mean (SD)	Median	IQR	z-Value	*p*-Value
Age (years)			49.85 (9.59)		46.77 (10.56)	45	18.25		52.93 (7.48)	52	12	−2.29	0.012
Body mass index (m^2^/kg)			24.99 (2.96)		25.63 (3.09)	25.25	3.29		24.35 (2.72)	23.92	2.90	1.75	0.094
Marital status	Married	44 (73%)		21 (70%)				23 (77%)					0.359
	Other	16 (27%)		9 (30%)				7 (23%)					
Job status	full time	48 (80%)		25 (83%)				23 (77%)					0.527
	part time	12 (20%)		5 (17%)				7 (23%)					
Working hours per week			56.03 (10.40)		57.35 (8.99)	60.00	13.75		54.72 (11.66)	55.00	14.375		0.332
Night work		35 (58%)		18 (60%)				17 (57%)					1.000
Employment	Self-employed	20 (33%)		10 (33%)				10 (33%)					
	hospital	38 (63%)		19 (63%)				19 (63%)					
	Self-employed and hospital	2 (3.3%)		1 (3.3%)				1 (3.3%)					
Job satisfaction	Very dissatisfied	1 (1.7%)		1 (3.3%)				0 (0%)					<0.001
	dissatisfied	1 (1.7%)		1 (3.3%)				0 (0%)					
	Partly satisfied, partly dissatisfied	14 (23%)		14 (47%)				0 (0%)					
	satisfied	21 (35%)		11 (37%)				10 (33%)					
	Very satisfied	23 (38%)		3 (10%)				20 (67%)					
Medical specialty	Psychiatry	6 (10%)		2 (6.7%)				4 (13.3%)					0.181
	Cardiology	3 (5%)		1 (3.3%)				2 (6.7%)					
	Internal medicine	20 (33%)		12 (40%)				8 (27%)					
	Oncology	4 (6.7%)		0 (0%)				4 (13%)					
	Surgery	11 (18.3%)		4 (13.3%)				7 (23.3%)					
	Neurology	3 (5%)		2 (6.7%)				1 (3.3%)					
	Other	13 (22%)		9 (30%)				4 (13.3%)					

**Table 2:** Descriptive statistics of Maslach burnout inventory, Narcissism Inventory TS (NI-2O), Effort–Reward Imbalance (ERI), and Perceived Stress Scale (PSS-4).

Variables		Total Sample, n = 60	Burnout, n = 30			Control, n = 30				
		Mean (SD)	Mean (SD)	Median	IQR	Mean (SD)	Median	IQR	z-Value	*p*-Value
Maslach Burnout Inventory (MBI)	Total score	1.68 (1.11)	2.68 (0.57)	2.62	0.91	0.68 (0.33)	0.71	0.50	6.65	<0.001
	Emotional Exhaustion	19.53 (12.78)	31.13 (5.84)	30.50	8.75	7.93 (4.43)	7.00	6.75	6.66	<0.001
	Depersonalization	8.05 (7.26)	13.77 (6.08)	12.00	8.75	2.33 (1.67)	2.00	2.00	6.45	<0.001
	Personal accomplishment	8.68 (5.58)	12.43 (4.61)	12.00	6.75	4.93 (3.6)	5.00	5.00	5.45	<0.001
Narcissism Inventory (NI20)	Threatened Self	1.55 (0.54)	1.87 (0.56)	1.71	0.71	1.25 (0.30)	1.14	0.29		<0.001
Perceived Stress Scale Sum Score (PSS-4)		4.66 (3.10)	6.45 (2.73)	7.00	4.00	2.93 (2.39)	3.00	2.75		<0.001
Effort–Reward Imbalance Questionnaire (ERI)	Effort–Reward Ratio	1.33 (1.22)	1.84 (1.54)	1.46	0.74	0.84 (0.41)	0.76	0.50	3.477	0.002
	Effort Subscale	6.36 (2.22)	7.69 (1.37)	8.00	2.00	5.01 (2.13)	5.00	2.00		<0.001
	Reward Subscale	13.53 (3.80)	11.97 (3.89)	12.00	6.00	15.03 (3.09)	15.00	4.00		0.001
	Overcommitment Subscale	8.48 (3.78)	10.68 (2.88)	11.00	4.25	6.43 (3.37)	7.00	4.00		<0.001

**Table 3:** Linear regression with outlier.

Coefficients:	Estimate	Std. Error	z-Value	*p*	CI Lower	CI Upper
(Intercept)	1.32	0.64	2.08	0.04 *	0.045	2.60
Narcissism Subscale “Threatened self”	1.10	0.21	5.14	<0.001 ***	0.669	1.52
PSS-4 Sum score	0.04	0.038	1.09	0.28	−0.035	0.117
Effort–Reward Ratio	0.05	0.08	0.68	0.50	−0.106	0.216
Age	−0.35	0.10	−3.47	0.001 **	−0.055	−0.015

CI Lower = lower bound of the 95% confidence interval, CI Upper = upper bound of the 95% confidence interval, * = *p* = 0.01, ** = *p* = 0.001, *** = *p* = 0

**Table 4:** Linear regression without outlier.

Coefficients:	Estimate	Std. Error	z-Value	*p*	CI Lower	CI Upper
(Intercept)	0.71	0.66	1.07	0.288	−0.615	2.03
Narcissism Subscale “Threatened self”	1.06	0.20	5.16	<0.001 ***	0.646	1.47
PSS-4 Sum score	0.03	0.04	0.68	0.498	−0.049	0.099
Effort–Reward Ratio	0.42	0.17	2.49	0.016 *	0.081	0.756
Age	−0.028	0.01	−2.84	0.006 **	−0.049	−0.008

CI Lower = lower bound of the 95% confidence interval, CI Upper = upper bound of the 95% confidence interval, * = *p* = 0.01, ** = *p* = 0.001, *** = *p* = 0

**Table 5:** Generalized linear model for analyzing the threatened self narcissism subscale as a predictor of burnout.

Coefficients:	Estimate	Odds Ratio	Std. Error	z-Value	*p*	CI Lower	CI Upper
(Intercept)	7.36	-	3.26	2.26	0.024 *	0.819	13.900
Narcissism Subscale “Threatened self”	−2.62	0.07	1.24	−2.12	0.034 *	−5.110	−0.140
PSS-4 Sum score	−0.33	0.72	0.18	−1.82	0.068	−0.685	0.032
Effort–Reward Ratio	−2.30	0.10	0.96	−2.39	0.017 *	−4.220	−0.374
Age	0.02	1.02	0.05	0.34	0.733	−0.080	0.113

* = *p* = 0.01.

## Data Availability

The data sets used and analyzed during the current study are available from the corresponding author upon reasonable request. Data cannot be publicly disclosed due to privacy and ethical restrictions.

## Abbreviations

The following abbreviations are used in this manuscript:

CnS	Classic narcissistic self
DP	Depersonalization
EE	Emotional exhaustion
ERI	Effort–Reward Imbalance
HS	Hypochondriac self
IS	Idealistic self
MBI	Maslach Burnout Inventory
NI-20	Narcissism Inventory
PA	Personal accomplishment
PHQ-9	Patient Health Questionnaire
PSS4	Perceived Stress Scale
TS	Threatened self
